# Editorial: Omic technologies, integrative methods and translational approaches in brain health and disease

**DOI:** 10.3389/fgene.2026.1927636

**Published:** 2026-07-16

**Authors:** Paolo Abondio, Francesco Bruno, Shaoyu Wang

**Affiliations:** 1 Department of Biology, University of Rome “Tor Vergata”, Rome, Italy; 2 Department of Human and Social Sciences, Faculty of Social and Communication Sciences, Universitas Mercatorum, Rome, Italy; 3 School of Dentistry and Medical Sciences, Charles Sturt University, Orange, NSW, Australia

**Keywords:** artificial intelligence, bioinformatics, healthspan, lifespan, neurodegeneration, neurodevelopment, neurotranscriptomics, translational neurology

Neurological research is entering a new era in which genetics, molecular biology, environmental factors, and computational technologies are converging to transform our understanding of brain health and disease. The four research topics examined here - APOE-mediated Alzheimer’s disease risk and reproductive aging, CNNM2-related neurodevelopmental disorders, selenium nanoparticle-based protection against liver-brain injury, and machine learning prediction of spinal cord neuronal states - represent diverse but interconnected advances. Together, they illustrate a central principle of modern neuroscience: neurological outcomes emerge from complex interactions among genes, metabolism, cellular mechanisms, and innovative technologies.

Alzheimer’s disease (AD) remains one of the greatest challenges in aging research, particularly in women, whose risk factors are not fully explained by traditional models. Bruno et al. investigated age at natural menopause (ANM), reproductive lifespan, and APOE genotype to address this gap by examining how hormonal history interacts with genetic susceptibility. The APOE gene is the strongest known genetic contributor to sporadic AD risk. The APOE ε4 allele is associated with increased disease risk, earlier onset, and altered responses to brain aging. However, the relationship between APOE and female reproductive biology has remained unclear. Estrogen has traditionally been viewed as neuroprotective because of its effects on synaptic function, mitochondrial health, and inflammation. Menopause, therefore, represents a major lifestage transition that may influence long-term brain vulnerability. Interestingly, the study challenges the assumption that longer estrogen exposure is always protective. Women with AD showed later menopause and longer reproductive lifespan than cognitively healthy controls, and both factors independently predicted increased AD risk. These associations were strongest among APOE ε4 carriers, suggesting that APOE may link reproductive aging with neurodegenerative processes. This finding highlights the importance of considering genetic background when interpreting hormonal influences on brain aging. In APOE ε4 carriers, prolonged ovarian hormone exposure may interact with lipid metabolism, inflammation, vascular pathways, or amyloid processing to increase vulnerability. By contrast, APOE ε3 appeared to reduce this effect. These results support a move toward personalized approaches to women’s brain health, integrating reproductive history and genetic profiles into risk assessment and prevention strategies.

While AD reflects complex interactions between aging and multiple biological factors, rare neurodevelopmental disorders reveal how specific genetic disruptions can alter brain development. Li et al. carried out a study of novel CNNM2 variants that expands understanding of hypomagnesemia, seizures and impaired intellectual development 1 (HOMGSMR1). Magnesium is essential for neuronal excitability, synaptic transmission, energy metabolism, and cellular signaling. The CNNM2 protein regulates magnesium transport across cell membranes, and disruption of this function can produce severe neurological consequences. Researchers identified two novel CNNM2 variants, p.E298del and p.P360R, and demonstrated that they alter protein localization. Instead of reaching the cell membrane, the abnormal proteins accumulate in the cytoplasm, forming aggregates and disturbing magnesium homeostasis. These variants also affect protein stability, suggesting that disease mechanisms involve not only altered gene function but also defects in cellular trafficking and degradation. The importance of this discovery extends beyond genetic characterization. Expanding knowledge of disease-causing variants improves diagnosis, genetic counseling, and the potential development of targeted therapies. CNNM2 research demonstrates how precision medicine begins with understanding the molecular foundations of neurological disease.

A different perspective on brain health emerges from research on the liver-brain axis, which emphasizes communication between organs. The brain does not operate independently from systemic biological processes. Metabolic disruption, inflammation, and oxidative stress originating in peripheral organs can contribute to neurological dysfunction of the brain. Chronic liver injury produces excessive reactive oxygen species (ROS) that damage proteins, lipids, mitochondria, and DNA. Because oxidative stress is also central to many neurodegenerative disorders, protecting the liver may represent an indirect strategy for maintaining brain health. Umapathy and Pan used mussel-derived selenium nanoparticles stabilized with bovine serum albumin to explore this possibility. Selenium plays a key role in antioxidant defense through selenoproteins, and nanoparticle-based formulations may improve its biological activity. In a zebrafish model exposed to copper sulfate-induced stress, these nanoparticles reduced oxidative damage, improved antioxidant enzyme activity, lowered lipid peroxidation, and protected liver tissue. This approach highlights the potential of nanomedicine to address systemic contributors to neurological decline. However, translation into clinical applications requires further evaluation of safety, dosage, and long-term biological effects. Nevertheless, the findings reinforce the growing recognition that preventing neurological diseases may require treating whole-body dysfunction rather than focusing exclusively on the brain.

Finally, Liu et al. challenged another major Frontier: artificial intelligence applied to cellular neuroscience. Single-cell transcriptomics has transformed our ability to study individual cells, but the complexity of these datasets requires advanced analytical approaches. ScnML, a machine learning model developed to predict spinal cord neuronal cell states, demonstrates how computational methods can accelerate discovery. The model achieved high accuracy in identifying neuronal subpopulations and revealed important marker genes that may not have been detected through conventional approaches. Such tools could have significant implications for precision medicine, spinal cord injury research, and neurorehabilitation. By identifying molecular signatures associated with specific neuronal states, machine learning can guide future therapeutic strategies and generate new biological hypotheses.

Together, these studies illustrate the rapidly evolving landscape of neuroscience: the future of brain medicine will depend on integrating these perspectives. Rather than studying genes, cells, organs and computational systems separately, neuroscience is moving toward a unified framework in which biological information is combined to improve prevention, diagnosis, and treatment. The next-generation of neurological breakthroughs will emerge from this integration of omic technologies, translational research and personalized medicine. This convergence reflects the broader scope of the present Research Topic, which spans epigenetics, the mobile genome, the gut-brain axis, and the development of novel statistical and bioinformatic methodologies, underscoring that no single omic layer can fully account for the complexity of brain health and disease. Several open questions remain: longitudinal data are needed to clarify the temporal relationship between reproductive aging and AD risk, the clinical translatability of nanoparticle-based neuroprotective strategies has yet to be established, and the generalizability of machine learning models such as ScnML across independent cohorts and species remains to be tested. Addressing these gaps will require sustained collaboration across genetics, systems biology, and computational science.

